# Two novel kindreds with autosomal recessive STAT2 deficiency

**DOI:** 10.70962/jhi.20260037

**Published:** 2026-06-30

**Authors:** Verena Kienapfel, Lotte Cresens, Lucy Bizien, Julia Vasconcelos, Marwa Chbihi, Margarida Guedes, António Marinho, Anneleen Hombrouck, Marjon Wouters, Dylan Laurens, Koji Nakajima, Jean-Laurent Casanova, Jacinta Bustamante, Shen-Ying Zhang, Paul Bastard, Leen Moens, Isabelle Meyts, Giorgia Bucciol

**Affiliations:** 1 Laboratory of Inborn Errors of Immunity, Department of Microbiology, Immunology and Transplantation, KU Leuven, Belgium; 2 Laboratory of Human Genetics of Infectious Diseases, Necker Branch, INSERM U1163, Necker Hospital for Sick Children, Paris, France; 3 Imagine Institute, Paris Cité University, Paris, France; 4 Pathology, Immunology Department, University Hospital Center of Porto, Porto, Portugal; 5 Unit for Multidisciplinary Research in Biomedicine, Institute of Biomedical Sciences Abel Salazar, University of Porto, Porto, Portugal; 6 Pediatric Immunology-Hematology and Rheumatology Unit, Laboratory of Immunogenetics of Pediatric Autoimmune Diseases, Inserm U1163, Necker-Enfants Malades Hospital, Assistance Publique - Hôpitaux de Paris, Paris, France; 7 Pediatrics Department, Maternal-Infantile North Center, University Hospital Center of Porto, Porto, Portugal; 8 Medicine Department, University Hospital Center of Porto, Porto, Portugal; 9 St. Giles Laboratory of Human Genetics of Infectious Diseases, Rockefeller Branch, The Rockefeller University, New York, NY, USA; 10 Department of Pediatrics, Necker Hospital for Sick Children, Assistance Publique - Hôpitaux de Paris, Paris, France; 11 Howard Hughes Medical Institute, New York, NY, USA; 12 Center for the Study of Primary Immunodeficiencies, Necker Hospital for Sick Children, Assistance Publique - Hôpitaux de Paris, Paris, France; 13 Department of Pediatrics, Division of Primary Immunodeficiencies, University Hospitals Leuven, Leuven, Belgium

## Abstract

STAT2 is a transcription factor in the type I/III interferon (IFN) antiviral response. Autosomal recessive STAT2 deficiency, reported in 12 kindreds from nine countries, underlies severe viral infections and hyperinflammation. We report three patients from two kindreds from Portugal and Algeria, respectively, with novel homozygous *STAT2* mutations. The three patients developed complications following live attenuated measles-mumps-rubella (MMR) vaccine, including hemophagocytic lymphohistiocytosis (HLH) in one patient, who responded to treatment with intravenous immunoglobulins (IVIG), steroids, and ruxolitinib (JAK-STAT inhibitor). The patients are now 36, 33, and 3 years old and healthy on prophylaxis with cotrimoxazole and itraconazole (P1), cotrimoxazole (P2), or IVIG (P3). The *STAT2* K490Qfs*41 and c.941+1G>T mutant alleles do not encode detectable and functional STAT2 proteins, and the patients’ primary cells do not respond to type I IFNs. STAT2 deficiency should be considered in patients with severe adverse reactions to MMR vaccination, and the use of targeted anti-inflammatory drugs like ruxolitinib should be further explored to control hyperinflammation.

## Teaser

Kienapfel et al. report the characterization of two novel mutations underlying STAT2 deficiency in three patients with viral infections and hyperinflammation following live viral vaccines. Beneficial use of ruxolinitib in one patient with vaccine-triggered hemophagocytic lymphohistiocytosis is also reported.

## Introduction

Type I interferons (IFNs) and, to a lesser extent, type II and type III IFNs, are crucial signaling molecules in antiviral defense. While type I IFNs (IFN-α/β) and type II IFNs (IFN-γ) are ubiquitously secreted, type III IFNs (IFN-λ) are only secreted by epithelial cells and dendritic cells (1, 2). The type I IFN receptor, a heterodimer composed by the two subunits IFNAR1 and IFNAR2, is ubiquitously expressed, while the type III IFN receptor, composed by the subunits IFNLR1 and IL10RB, is only expressed on epithelial cells, blood–brain barrier endothelial cells, and a subset of human leukocytes (plasmacytoid dendritic cells and B cells) (3, 4, 5). After binding to their receptors, type I and III IFNs trigger the phosphorylation of kinases constitutively associated to their receptors (TYK2 and JAK1) and the recruitment of signal transducer and activator (STAT) molecules (6, 7). Although seven STAT molecules are known, STAT1 and STAT2 are the main players in antiviral defense (8). After their recruitment, STAT1 and STAT2 are phosphorylated and form dimers. STAT1 homodimerizes and binds to γ-activated sequence elements in the promoter of IFN-stimulated genes (ISGs). While STAT1 can form homodimers, STAT2 can only bind to STAT1 to form heterodimers. The heterodimer combines with IRF9, and the resulting complex, heterotrimer ISG factor 3 (ISGF3), translocates to the nucleus. There, ISGF3 can bind to the promoter region of ISGs to induce antiviral response. In addition, STAT2 recruits ubiquitin-specific protease 18 (USP18) to IFNAR2. USP18 inhibits the function of IFNAR2 and downregulates the type I IFN response, thus preventing hyperactivation and mediating the resolution of the IFN response (7, 9, 10, 11, 12). Stimulation with type I and type III IFN induces mainly the formation of STAT1/STAT2 heterodimers, while stimulation with type II IFN (IFN-γ) induces the formation of STAT1 monomers (2).

Five loss-of-function (LOF) defects in genes connected to this signaling cascade have been reported. IFNAR1, IFNAR2, IRF9, and STAT2 deficiencies are characterized by susceptibility to viral infections, including those caused by live-attenuated vaccines (LAVs), while STAT1 deficiency causes a broader susceptibility to viral and mycobacterial infections (7, 9, 11, 13, 14, 15, 16, 17, 18, 19). The typical presentation of autosomal recessive (AR) STAT2 deficiency is indeed disseminated infection and encephalitis following administration of LAV. Hence, most patients are diagnosed early in life following vaccination with the measles-mumps-rubella (MMR) vaccine, although penetrance is not complete for this manifestation (16). Patients are also susceptible to severe infection with naturally occurring viral agents such as herpes simplex virus 1, Epstein-Barr virus, respiratory syncytial virus, influenza A, or severe acute respiratory syndrome coronavirus 2 (SARS-CoV-2) (7, 9, 11, 13, 16, 17, 20). In addition, systemic inflammation following infection is a typical clinical presentation of STAT2 deficiency and is triggered by uncontrolled viral proliferation and a prominent neutrophil and monocyte signature (16). 25 patients with AR STAT2 deficiency have been reported so far, with mortality reaching 35% in early childhood (Table 1 and Table 2) (16, 21, 22, 23).

**Table 1.** Genetic information of reported STAT20-deficient patients

Patient	Nucleotide change	Deduced amino acid change	MAF	Intron/Exon	CADD score	Predicted effect	In silico splicing prediction	Effect on STAT2 mRNA and protein	Reference
P1 and P2	c.1467_1468insC	K490Qfs*41	Private	Exon 17	24	Frameshift + stop-gain	​	Complete loss of expression of STAT2	Current report
P3	c.941+1G>T	–	Private	Intron 9	33	Splice mutation	Disrupted WT donor splice site	Complete loss of expression of STAT2	Current report
P4–P9	c.381+5G>C	NA	Private	Intron 4	11.71	Splice mutation	Disrupted WT donor splice site	Retention of introns 4 and 6, skipping of exons 16 and 17, nonsense-mediated decay, and complete loss of expression of STAT2	(16, 17)
P10 and P11	c.1836C>A	C612X	Private	Exon 20	37	Stop-gain	​	Complete loss of expression of STAT2	(24)
P12 and P13	c.1528C>T c.1576G>A	R510X NA	Private	Exon 17 Exon 16	35 33	Stop-gain Splice mutation	Disrupted WT donor splice site	cDNA nonsense-mediated decay and complete loss of expression of STAT2	(25)
P14 and P15	c.1883_1884del	V628fs*14	Private	Exon 21	34	Frameshift + stop-gain	​	Complete loss of expression of STAT2	(16)
P16–P18	c.988C>T	R330X	Private	Exon 10	36	Stop-gain	​	Complete loss of expression of STAT2	(16)
P19–P22	c.820C>T	Q274X	Private	Exon 9	34	Stop-gain	​	Complete loss of expression of STAT2	(16)
P23	c.1999C>T	R667X	Private	Exon 21	38	Stop-gain	​	Complete loss of expression of STAT2	(21)
P24	c.1209+1delG	NA	Private	Intron 13	24.1	Splice mutation	Disrupted WT donor splice site	Complete loss of expression of STAT2	(26)
P25 and P26	DelChr12:56360796-56352109/DelChr12:56355504-56348082	NA	Private	5′ upstream-intron 8 Exon 5-intron 19	NA	Large deletion	​	Complete loss of expression of STAT2	(16)
P27	c.633+2T>C	​	Private	Intron 7	​	Splice mutation	Disrupted WT donor splice site	Complete loss of expression of STAT2	(27)
P28	c.2053C>T c.1838C>T	Q685X S613F	Private	Exon 22 Exon 20	35 29.4	Stop-gain	​	Complete loss of expression of STAT2	(22)

MAF, minor allele frequency; WT, wild type.

**Table 2.** Summary of the clinical characteristics of reported STAT2-deficient patients

Patient	Genetic defect	Birth year	Sex	Symptom onset age	Bacterial infections	Viral infections	Cytopenia	Other	LAV	Complications after LAV	Outcome	Current treatment	Reference
P1	K490Qfs*41	1989	F	22 days	Recurrent infections	Viral meningitis	No	–	MMR	Hepatosplenomegaly, adenomegalies, and oral candida	Alive	Cotrimoxazole Itraconazole	Current report
P2	K490Qfs*41	1992	M	4 mo	*S. epidermidis*	Chickenpox	No	–	MMR	Fever, rash, conjunctivitis, seromucous rhinitis, cheilitis, pharyngitis, and edema (atypical Kawasaki disease)	Alive	Cotrimoxazole	Current report
P3	c.941+1G>T	2022	M	1 year	No	No	No	–	MMR	HLH	Alive	IVIG	Current report
P4	c.381+5G>C	1982	F	NA	No	NA	No	–	NA (positive serological tests)	NA	Alive	None	(17)
P5	c.381+5G>C	2006	F	2 years	No	Influenza A pneumonia, HSV stomatitis, asymptomatic EBV, and mild SARS-CoV-2	No	–	MMR	Disseminated measles with pneumonia	Alive	Acyclovir	(17)
P6	c.381+5G>C	2007	M	2 mo	No	NA	No	–	No	–	Died at 2 mo	–	(17)
P7	c.381+5G>C	2006	F	1 year	No	Several URTI, chickenpox	No	–	MMR	Prolonged fever, rash, profound sensorineural hearing loss	Alive	Acyclovir	(17)
P8	c.381+5G>C	2008	F	NA	No	Several URTI, chickenpox	No	Asthma, urticaria	No	–	Alive	Acyclovir cetirizine	(17)
P9	c.381+5G>C	2014	F	NA	No	Several URTI	No	Atypical Kawasaki disease	No	–	Alive	Acyclovir	(16)
P10	C612X	2009	M	1 year	No	NA	No	–	MMR	Disseminated measles with systemic inflammation, atypical Kawasaki disease, and meningoencephalitis	Alive	SCIG levetiracetam, topiramate, lacosamide, and omeprazole	(24)
P11	C612X	2010	F	1 year	No	No	Yes	–	MMR	Disseminated measles with systemic inflammation, cytopenia, coagulopathy, and sepsis-like	Alive	SCIG	(24)
P12	R510X/c.1576G>A	1995	M	3 mo	Otitis, pneumonia, mycoplasma pneumonia	Recurrent severe viral infections, RSV, adenovirus, and enterovirus	Yes	–	MMR	Disseminated measles with systemic inflammation and atypical Kawasaki disease	Died at 7 years	–	(25)
P13	R510X/c.1576G>A	2004	F	6 mo	Otitis, pneumonia	Recurrent viral infections, influenza A pneumonia, severe chickenpox, severe EBV, enterovirus B virus meningitis, and mild SARS-CoV-2	Yes	​	MMR	Disseminated measles with systemic inflammation, pneumonia, and hepatitis	Alive	None	(25)
P14	V628fs*14	2002	F	1 year	Otitis	Enterovirus B meningitis, severe SARS-CoV-2, and pneumonia	No	​	MMR	Disseminated measles with systemic inflammation, atypical Kawasaki disease, and meningitis	Alive	Cetirizine, inhaled fluticasone-salmeterol	(16)
P15	V628fs*14	2012	F	9 mo	No	RSV, rotavirus, influenza A pneumonia, and enterovirus enteritis	No	​	MMR	MA	Died at 5 years	–	(16)
P16	R330X	1998	F	3 mo	Pneumonia, urinary tract infections	Recurrent URTI	No	Bronchitis, hypo-IgA	MMR, BCG	No	Alive	None	(16)
P17	R330X	2015	F	5 mo	Pneumonia	Chickenpox	No	–	MMR, BCG	No	Alive	IVIG, cotrimoxazole	(16)
P18	Sibling to P16 and P17	1996	F	2 years	Pneumonia	Severe HSV stomatitis and encephalitis	NA	–	MMR, BCG	NA	Died at 2 years	–	(16)
P19	Q174X	2014	M	9 mo	Bacterial pneumonia during ARDS due to influenza pneumonia	Influenza A with ARDS, enterovirus, HSV stomatitis, and keratitis	No	Sensorineural hearing loss	MMR	Mumps, systemic inflammation, atypical Kawasaki disease, and sensorineural hearing loss	Died at 5 years	–	(16)
P20	Sibling to P19	2008	F	1 year	Recurrent infections	Influenza A pneumonia	NA	–	MMR, VZV	NA	Died at 2 years	–	(16)
P21	Q274X	2016	F	7 mo	Pneumonia	Asymptomatic EBV	No	–	Measles	Clinical measles	Alive	IVIG	(16)
P22	Sibling to P22	NA	M	9 mo	–	–	NA	–	Measles, MMR	Prolonged fever, clinical mumps	Died at 5 years	–	(16)
P23	R667X	2017	F	9 mo	Recurrent otitis and pneumonia	Influenza A with ARDS, several URTI, coronavirus HKU1, rhinovirus, human metapneumovirus, HHV6, parainfluenza, and mild SARS-CoV-2	Yes (during HLH episode)	–	MMR, VZV	Disseminated VZV, systemic inflammation, and HHV6-triggered HLH	Alive	None	(21)
P24	c.1209+1delG	2017	M	1 year	Recurrent otitis	Norovirus, enterovirus, HHV6, CMV, and influenza A	Yes (during HLH episode)	–	MMR, VZV	Systemic inflammation, HLH, and mumps meningitis	Alive	IVIG	(26)
P25	DelChr12:56360796-56352109/DelChr12:56355504-56348082	2009	F	Birth	Recurrent pneumonia	EBV, asymptomatic SARS-CoV-2, several URTI	No	Oral ulcers, appendicitis	No	–	Alive	IVIG, cotrimoxazole	(16)
P26	Sibling to P25	2004	M	4 mo	–	Severe HSV stomatitis, recurrent severe URTI	NA	–	No	–	Died at 10 mo	–	(16)
P27	c.633+2T>C	NA	F	2 mo	–	URTI, MIS-C associated to SARS-CoV-2 infection, rhinovirus	Yes	MIS-C	MMR	MMR-associated HLH, febrile illness with seizure	Alive	Anakinra	(27)
P28	Q685X S613F	NA	M	2 years	–	Recurrent severe influenza pneumonia, critical SARS-CoV-2	NA	–	MMR	Aseptic meningitis and Kawasaki disease after MMR vaccination	Alive	NA	(22)

ARDS, acute respiratory distress syndrome; BCG, Bacille Calmette-Guerin; CMV, cytomegalovirus; EBV, Epstein-Barr virus; F, female; HHV6, human herpesvirus 6; HSV, herpes simplex virus; M, male; MIS-C, multi-inflammatory syndrome in children; mo, months; RSV, respiratory syncytial virus; URTI, upper respiratory tract infections; VZV, varicella-zoster.

Here we describe two unrelated kindreds harboring two novel variants in *STAT2*, underlying AR STAT2 deficiency.

## Results

### Biallelic *STAT2* variants in 2 kindreds

**Figure 1.**
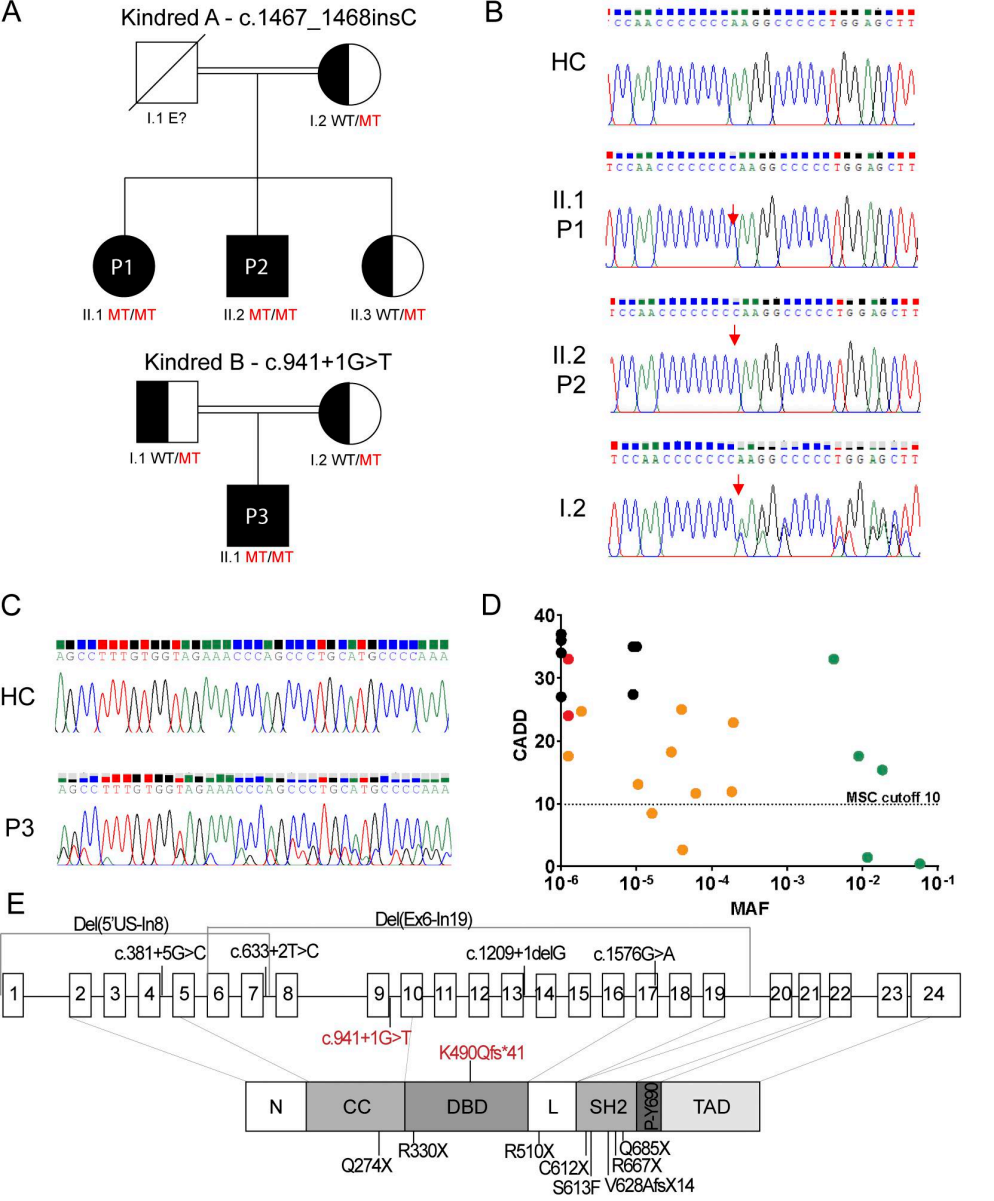
**Novel *STAT2* variants in two kindreds. (A)** Pedigrees of two unrelated kindreds with *STAT2* variants. Double lines connecting parents indicate consanguinity. Filled symbols indicate individuals with homozygous mutations, and half-filled symbols indicate carriers of heterozygous mutations. E? indicates unknown genotype. **(B)** Sanger sequencing results for c.1467_1468insC in an unrelated healthy control (HC), a related carrier (I.2), P1 (II.1), and P2 (II.2). **(C)** Sanger sequencing results for cDNA of the beginning of exon 10 of an unrelated healthy control (HC) and P3. **(D)** Population genetics of two novel *STAT2* variants compared to homozygous coding missense *STAT2* mutations from gnomAD and known pathogenic variants from the literature. Green: gnomAD variants predicted benign; orange: gnomAD variants of uncertain significance; black: eight previously reported pathogenic biallelic *STAT2* variants; red: novel *STAT2* variants K490Qfs*41 and c.941+ 1G>T. MAF, minor allele frequency; MSC, mutation significance cutoff. **(E)** Schematic illustration of the *STAT2* gene and the STAT2 protein with its domains. Previously reported *STAT2* variants are indicated in black, novel variants in red. N, N-terminal domain; CC, coiled domain; DBD, DNA-binding domain; L, linker domain; SH2, Scr homology two domain; P-Y690, tyrosine phosphorylation site; TAD, transcriptional activation domain.

**Figure S1.**
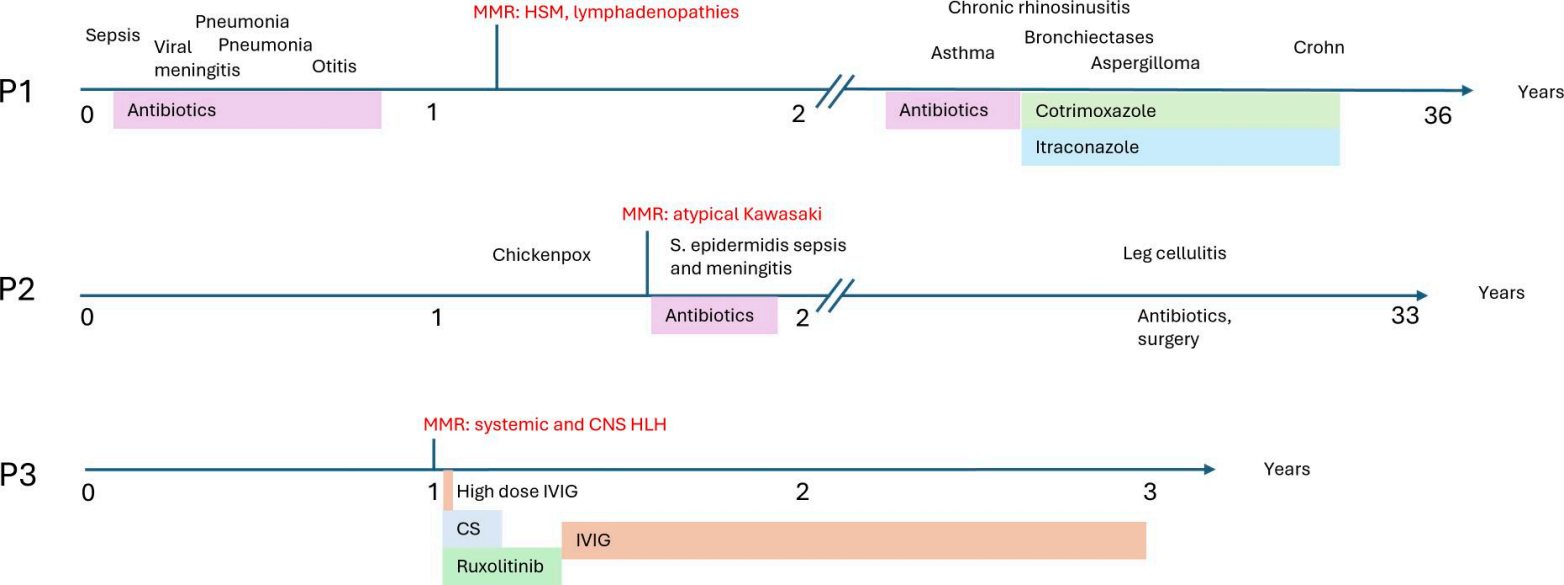
**Clinical presentation of** **the patients. CNS, central nervous system; CS, cortico-steroids; HLH, hemophagocytic lymphohistiocytosis; HSM, hepatosplenomegaly; IVIG, intravenous immunoglobulins; MMR, measles-mumps-rubella vaccine.**

We studied three patients from two kindreds carrying biallelic STAT2 variants (Fig. 1 A and Fig. S1). Patients 1 (P1) and 2 (P2) are siblings born to healthy consanguineous parents (first cousins) from Portugal. They both presented with frequent recurrent infections and hyperinflammatory symptoms. Briefly, P1 suffered recurrent severe infections since the age of 22 days, including sepsis, viral meningitis, multiple episodes of pneumonia, purulent otitis, and gastroenteritis. 15 days after MMR vaccination, P1 presented with hepatosplenomegaly, generalized lymphadenopathies, and oral candidiasis. She developed asthma, chronic rhinosinusitis, localized bronchiectases with frequent chest infections necessitating antibiotics, and a pulmonary aspergilloma. She was treated with cotrimoxazole and itraconazole as prophylaxis. At the age of 31 years, she developed a chronic anal fissure and terminal ileitis due to Crohn’s disease was diagnosed.

P2 developed varicella at the age of 15 mo, which resolved without complications. Nine days after MMR vaccination, P2 was hospitalized with a working diagnosis of atypical Kawasaki disease characterized by fever, rash, conjunctivitis, rhinitis, cheilitis, pharyngitis, and edema of the extremities, complicated by a *Staphylococcus epidermidis* sepsis and meningitis. Notably, he had persistent fever for 41 days during this infectious episode. He had an episode of cellulitis of the leg requiring surgical debridement at the age of 31 years old. He was treated with prophylactic cotrimoxazole. Both patients had normal serum levels of IgA, IgM, IgG, and IgE. Specific IgG antibodies against pneumococcal polysaccharides, tetanus toxoid, and rubella were in normal range. Moreover, normal complement activity and oxidative burst were observed.

P3 was born to nonconsanguineous parents of Algerian origin and presented at the age of 12 mo with a severe adverse reaction following MMR vaccine with polymorphic cutaneous rash and fever. On admission, she had bicytopenia (anemia and thrombocytopenia), hyperferritinemia (3,400 µg/L), hypofibrinogenemia (0.4 g/L), and hepatosplenomegaly. Her immunophenotyping showed markedly increased effector memory (EM) CD8^+^ T cells. A lumbar puncture revealed lymphocytic hypercellularity but was negative for hemophagocytosis, while a bone marrow aspirate showed a normocellular marrow enriched in macrophages and with ongoing hemophagocytosis. A brain MRI was performed and showed diffuse parenchymal atrophy associated with a washed-out appearance and T2/FLAIR hyperintensity in the deep white matter and mild signal abnormality in the lenticular nuclei with increased cerebral blood flow, compatible with central hemophagocytic lymphohistiocytosis (HLH). Systemic and central nervous system HLH was diagnosed based on the HLH-2024 criteria (28). She was treated with one course of high-dose intravenous immunoglobulins (IVIG), steroids, and ruxolitinib (20 mg/m^2^/day) for 2 mo with complete resolution of the cytopenia, normalization of fibrinogen, triglycerides, and cellularity in the cerebrospinal fluid, reduction of ferritin levels, and resolution of inflammation and the other HLH symptoms.

Whole-exome sequencing was performed on P1, P2, and their healthy mother to search for candidate genetic variants, testing the AR inheritance model. The variants were then filtered according to their frequency (minor allele frequency <0.01) and quality (read depth > 10). As a result, a homozygous c.1467_1468insC (K490Qfs*41) STAT2 variant (NM_005419.4, 12:56349033-5649033) was identified in P1 and P2, which was further confirmed by Sanger sequencing (Fig. 1, A and B). The healthy mother and healthy sibling were heterozygous, and no material was available from the father (Fig. 1, A and B). The mutation was predicted to be pathogenic in silico (combined annotation dependent depletion [CADD] score: 24, mutation significance cutoff [MSC] 10) and to lead to an early stop interrupting the translation (ExPASy, Swiss-Prot Group, https://web.expasy.org/translate/). Five other homozygous rare nonsynonymous variations were also identified, but none of them resided in known inborn error of immunity (IEI)–related genes (Table S3).

A novel private homozygous essential splice variant in the *STAT2* gene (12:56351291, c.941+1G>T) was found in P3 by a next generation sequencing–based panel, consisting of 500 known IEI-related genes. This variant was predicted to be pathogenic in silico (CADD score: 33, SpliceAI: donor loss). Both parents were heterozygous carriers of this variant (Fig. 1, A–C). Upon diagnosis of STAT2 deficiency, IVIG substitution was started and ruxolitinib was discontinued. The population genetics and position in *STAT2* of the two novel variants relative to the other reported *STAT2* mutations are shown in Fig. 1, D and E.

### Both K490Qfs*41 and c.941+1G>T *STAT2* variants lead to loss of STAT2 expression

**Figure 2.**
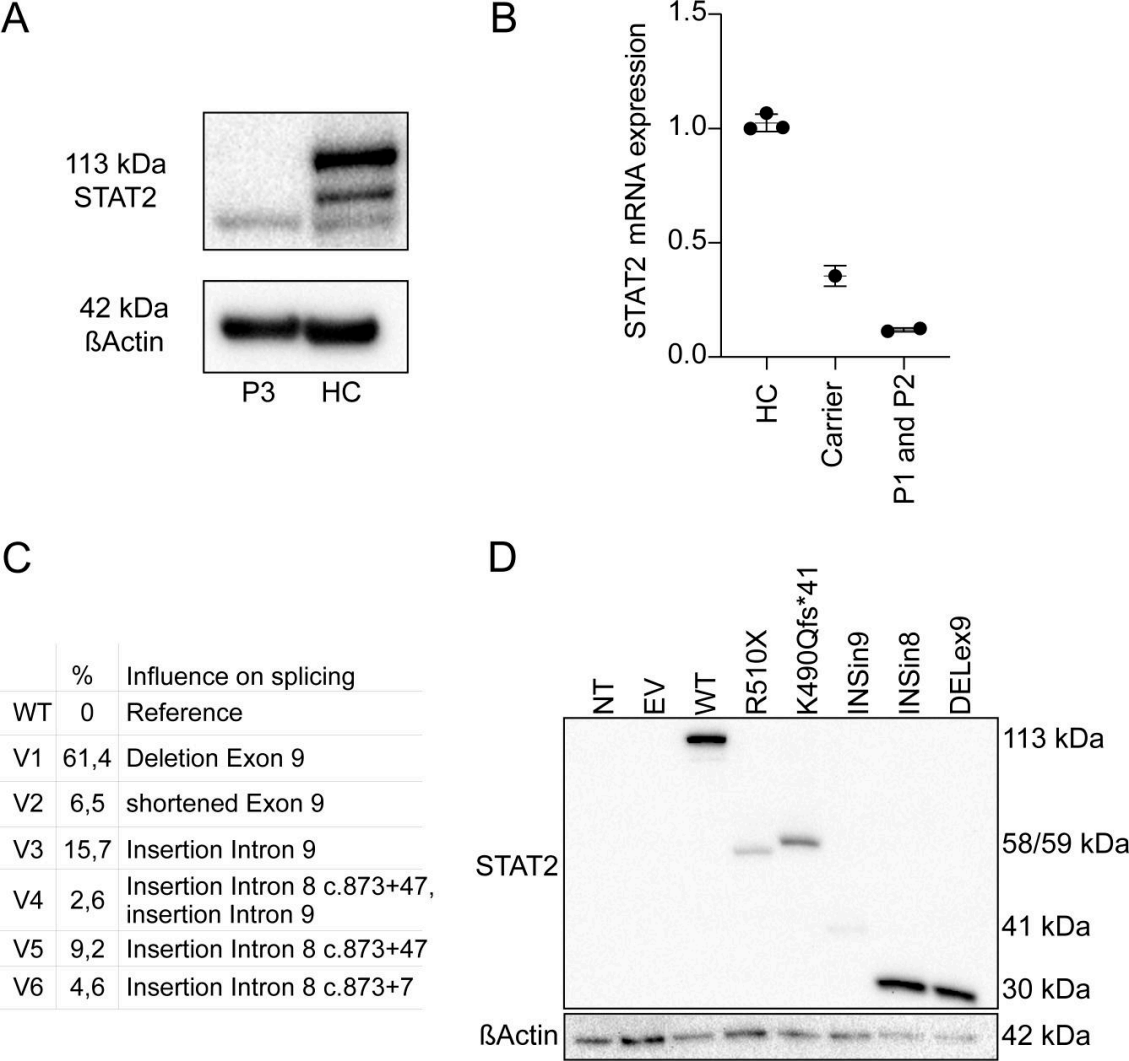
**Impact of the variants on STAT2 mRNA and protein expression. (A)** Immunoblot of STAT2 in PBMCs of P3 (*N* = 1). **(B)***STAT2* mRNA expression of primary fibroblasts of P1 and P2 as well as a related carrier and three unrelated healthy controls (HC). Each dot represents the mean value of three independent experiments for a single individual. **(C)** pJet cloning results of 182 analyzed clones of genetic material of P3. **(D)** Immunoblot of STAT2 in HEK293T cells expressing the different STAT2 variants. NT (non-transfected), EV (empty vector), WT (wild-type), R510X (positive control), K490Qfs*41 (variant in P1 and P2), and P3’s splice variants: INSin9, INSin8, and DELex9. A representative blot from three experiments is shown. Source data are available for this figure: SourceData F2.

**Figure 3.**
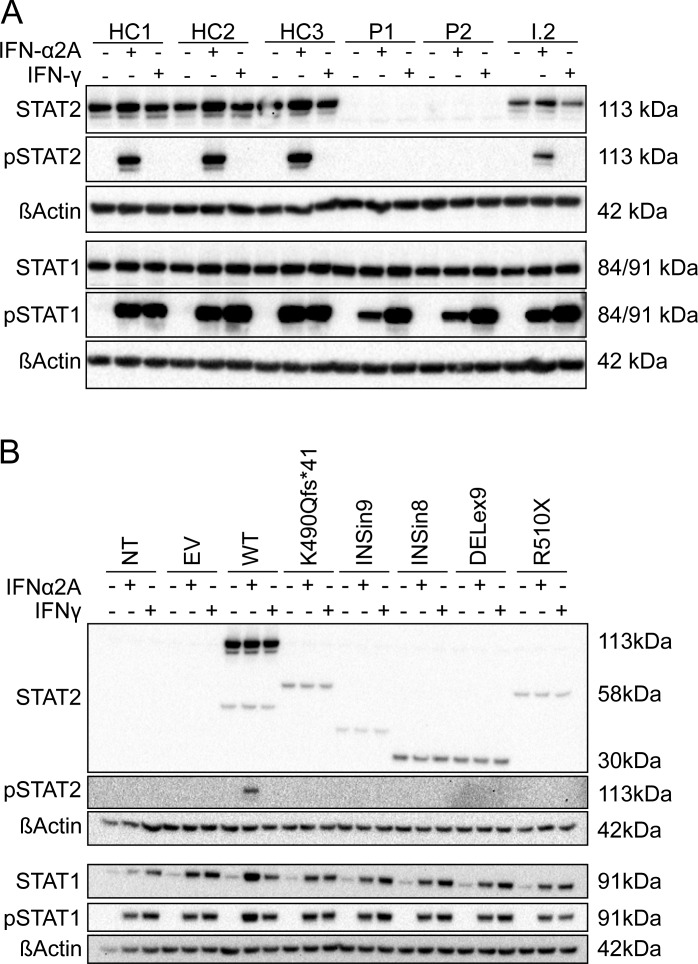
**The *STAT2* variants impair STAT2 protein expression and phosphorylation. (A and B)** Transfected HEK293T cells (B) and primary fibroblasts (A) were stimulated with 10,000 IU/ml IFN-α2A and 1,000 IU/ml IFNγ for 30 min. **(A)** Immunoblots of whole-cell extracts from primary fibroblasts of P1 and P2, their mother (I.2), and three healthy controls (HC) show an absent STAT2 expression in P1 and P2, as well as a lower expression in their mother (I.2). A representative blot from two experiments is shown. **(B)** Immunoblots of whole-cell extracts from HEK293T cells transfected with the following *STAT2* variants: NT (non-transfected), EV (empty vector), WT (wild-type), R510X (positive control), K490Qfs*41 (variant in P1 and P2), and P3’s splice variants: INSin9, INSin8, and DELex9. A representative blot from three experiments is shown. Source data are available for this figure: SourceData F3.

**Figure S2.**
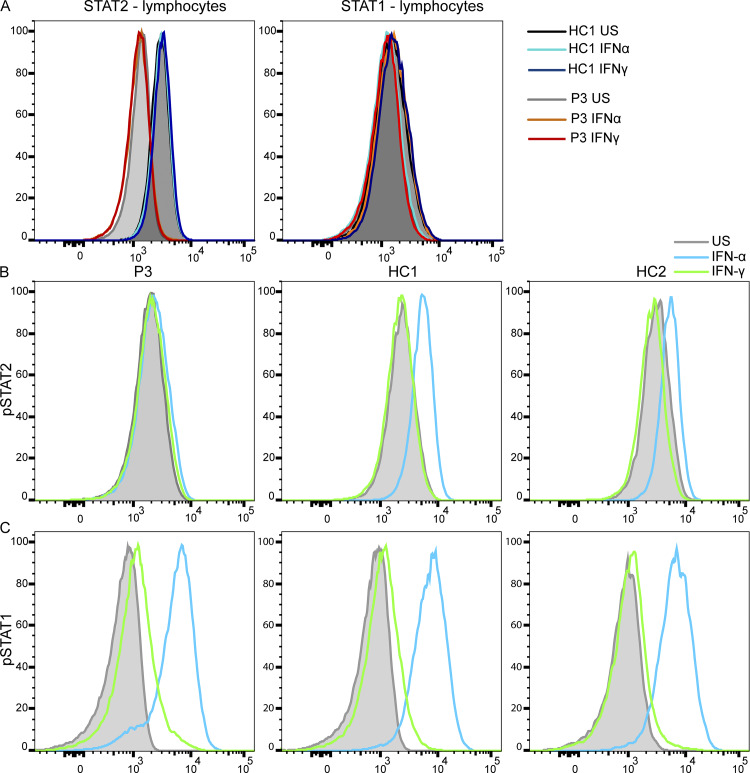
**Expression of phosphorylated STAT2 (pSTAT2), STAT2, pSTAT1, and STAT1 in lymphocytes derived from P3 and two healthy controls (HC).** **(A–C)** (A) STAT2 and STAT1, (B) pSTAT2 and (C) pSTAT1 expression in lymphocytes after treatment with 10.000 IU/mL IFNγ and 10.000 IU/ml IFNα for 25 min.

Full-length STAT2 protein expression was absent in patients’ primary cells (peripheral blood mononuclear cells [PBMCs] for P3 and fibroblasts for P1 and P2), as measured by immunoblot (Fig. 2 A and Fig. 3 A). Quantitative PCR (qPCR) revealed complete absence of *STAT2* mRNA in primary fibroblasts of P1 and P2 carrying the K490Qfs*41 variant (Fig. 2 B). P3’s variant c.941+1G>T is located within a splice donor site, suggesting a possible effect on the splicing of *STAT2* mRNA. Sequencing of P3’s cDNA after pJet cloning identified six different splice variants but no wild-type *STAT2* (Fig. 2 C). The three most frequent transcripts, namely the deletion of exon 9 (DELex9), the insertion of intron 9 (INSin9), and the insertion of the first 47 nucleotides of intron 8 (INSin8), as well as P1 and P2’s K490Qfs*41 variant, were used to generate plasmids via site-directed mutagenesis and overexpressed in HEK293T cells. The variant R510X was included as a known LOF stop-mutation (25) Variable amounts of truncated STAT2 proteins of different size were detected for each variant by immunoblot (Fig. 2D). Finally, FACS analysis confirmed the absence of STAT2 protein expression in P3-derived T cell blasts (Fig. S2).

### The *STAT2* variants result in loss of STAT2 phosphorylation upon stimulation with IFNα

The phosphorylation of STAT2 and STAT1 was induced through 30 min of IFNα and IFNγ stimulation, respectively, both in P1 and P2’s primary fibroblasts (Fig. 3 A) and in HEK293T cells transfected with plasmids containing the STAT2 variants K490Qfs*41, DELex9, INSin9, and INSin8 (Fig. 3 B). STAT1 phosphorylation was normal for all variants and controls after stimulation with IFNα and IFNγ (Fig. 3, A and B). None of the patients’ variants in the HEK overexpression system showed any phosphorylation of STAT2 after stimulation with IFNα, while this was normal in healthy controls (Fig. 3 B). Absence of STAT2 phosphorylation after treatment with IFNα for 30 min was also shown in primary fibroblast of P1 and P2 (Fig. 3 A) and in T cell blasts of P3 (Fig. S2, showing also normal STAT1 phosphorylation in the same cells), while it was preserved in P1 and P2’s carrier mother (Fig. 3 A).

### The *STAT2* variants result in lack of ISG induction following stimulation with IFNα

**Figure 4.**
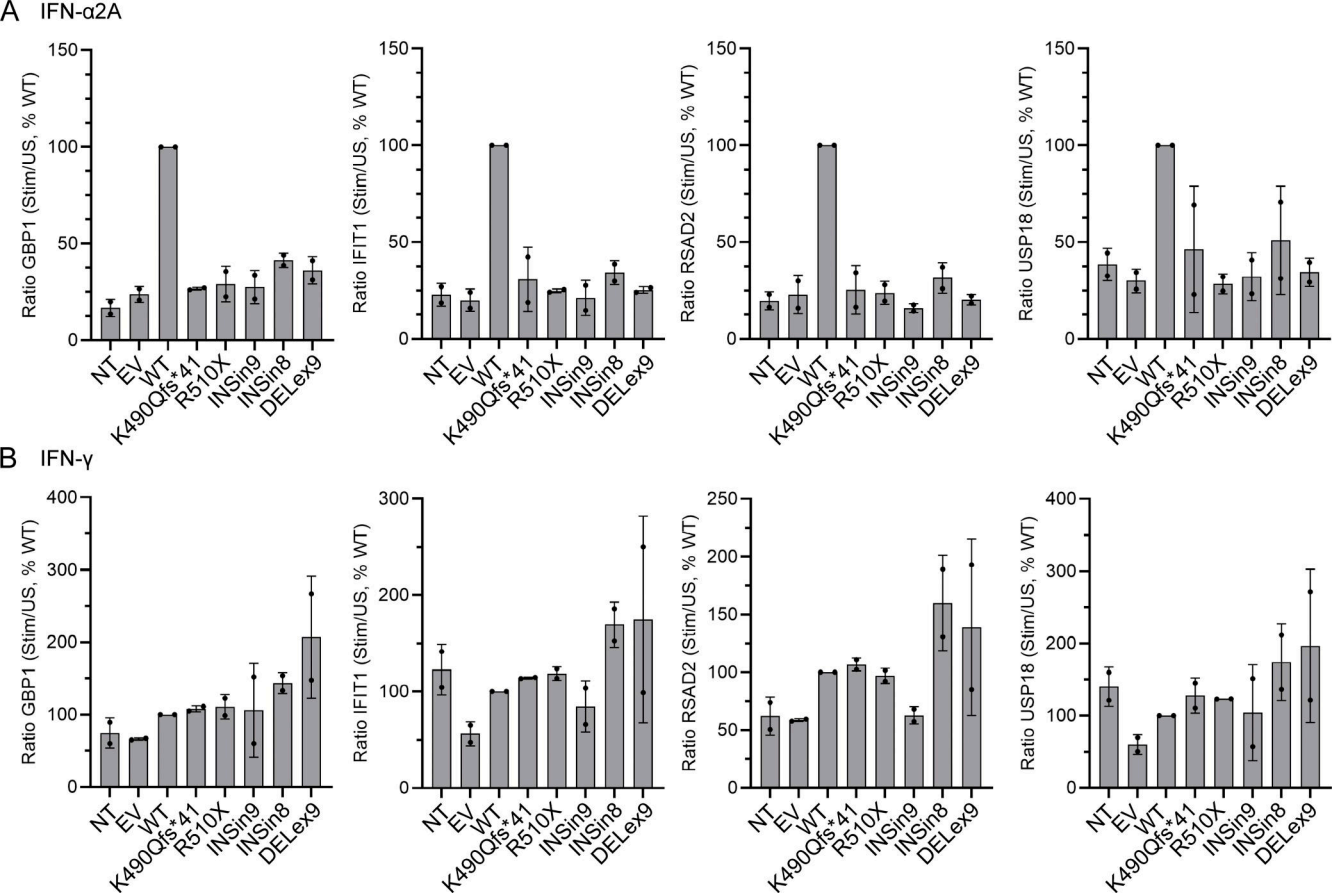
**The STAT2 variants impair downstream type I IFN signaling. (A and B)** Transcription levels of *GBP1*, *IFIT1*, *RSAD2*, and *USP18* assessed by RT-qPCR on STAT2-deficient U6A fibrosarcoma cells transfected with empty vector (EV), WT *STAT2*, R510X (positive control), K490Qfs*41, INSin9, INSin8, and DELex9. Cells were pretreated with 10,000 IU/ml IFN-α2A (A) or 1,000 IU/ml IFN-γ (B) for 6 h. A representative blot from two experiments is shown.

We next measured the induction of ISGs expression after 6 h of IFNα and IFNγ stimulation in STAT2-deficient U6A fibrosarcoma cells transfected with plasmids containing the STAT2 variants K490Qfs*41, DELex9, INSin9, and INSin8. The upregulation of the ISGs *RSAD2*, *IFIT1*, *GBP1*, and *USP18* was identified by qPCR. The variant R510X was included as a known LOF stop-mutation (25). The studied *STAT2* variants failed to induce ISG transcription following type I IFN stimulation (Fig. 4 A). In contrast, stimulation with IFNγ led to a normal upregulation of transcription of ISGs (Fig. 4 B).

## Discussion

We report two novel homozygous LOF mutations in *STAT2* leading to AR STAT2 deficiency in two unrelated families from Portugal and Algeria. Both mutations (K490Qfs*41 in P1 and P2 and c.941+1G>T in P3) lead to the complete absence of full-length STAT2 protein in the patients. Functional analysis of these variants show that they lead to absent or truncated proteins that undergo degradation and are not functional, as they do not support STAT2 phosphorylation and the induction of ISG transcription in response to IFNα.

All three patients reported in this study experienced life-threatening infection with live-attenuated MMR. Severe reaction to LAVs is a key presentation of AR STAT2 deficiency. In a recent study, 12 out of 18 patients with STAT2 deficiency experienced infectious and/or inflammatory complications, including HLH and Kawasaki-like disease, after LAV administration (16). HLH as complication of LAV administration was also described in IFNAR1, IFNAR2, IRF9, and STAT1 deficiency (26, 27, 29) and has recently been reported in another STAT2 deficient patient after MMR vaccination (27). Interestingly, this patient also suffered from multisystem inflammatory syndrome in children after SARS-CoV-2 infection, despite being vaccinated against it (27). In this study, P3 presented with HLH after MMR vaccination and was treated with immune suppression including ruxolitinib, an inhibitor of JAK1 and JAK2 (Jakinib). The patient’s cytopenia resolved during ruxolitinib treatment, although it was discontinued after the diagnosis of STAT2 deficiency because of the risk of viral infections. Nevertheless, its successful and safe use raises the question whether patients suffering from hyperinflammation in the context of type I IFN defects could indeed benefit from Jakinibs or other targeted anti-inflammatory treatments. Hyperinflammation, especially in the form of HLH, has been extensively described in patients with STAT2, IFNR1, and IRF9 deficiency (1, 9, 12, 16, 21, 26, 27). A proposed underlying mechanism could be a prolonged type I IFN signaling due to insufficient negative regulation by STAT2 and USP18, although we previously showed an increased tumor necrosis factor (TNF)/nuclear factor κ-B (NF-κB) and JAK/STAT3 signaling and roles for circulating monocytes, neutrophils, and CD8 EM cells in inflammation in patients with STAT2 or related deficiencies (9, 16). We speculate that ruxolitinib, acting directly on the JAK–STAT complexes, was effective in interrupting this proinflammatory cascade and assisted in the downregulation of this abnormal response.

In young patients with severe viral infections and/or complications after LAVs, STAT2 deficiency should be excluded. Since STAT2 deficiency is fatal in 35% of patients in early childhood (7, 16, 30), early genetic testing is recommended. LAVs are to be avoided in STAT2-deficient patients, and early intervention in case of viral infections is crucial. The mortality rate seem to decrease with age, probably due to compensation from the adaptive immune system and other innate immune components that seem to rely less on type I IFN for their development and function, such as natural killer cells (31). Nevertheless, exposure to novel viruses can still cause severe and even fatal outcomes in adulthood, as the recent SARS-CoV-2 pandemic demonstrated (16, 22, 32, 33). Despite a still significant mortality after LAV and during acute viral infections, STAT2-deficient patient experience relatively few life-threatening infections or other complications, not warranting the risk of curative treatments like hematopoietic stem cell transplantation. They are usually managed with antiviral and supportive treatment during infectious/inflammatory episodes and sometimes with prophylactic acyclovir and immunoglobulin substitution. The use of ruxolitinib as a targeted anti-inflammatory drug during acute episodes of hyperinflammation/HLH could be an interesting addition to the management of STAT2-deficient patients, as successfully demonstrated in P3. An alternative could be emapalumab, a monoclonal antibody that has been approved for the treatment of primary HLH (34, 35). Targeting IFN-γ specifically, emapalumab could counteract STAT1-mediated hyperinflammation while presenting a safer profile in the context of viral infections compared to the broader action of ruxolitinib. Nevertheless, monoclonal/immunomodulatory agents should be tested with caution, ideally in the context of a multicenter clinical study with protocols to assess efficacy and safety.

Overall, our case report contributes to a better understanding of STAT2 deficiency and human antiviral immunity. Our patients’ presentations confirm once again that the phenotypic spectrum of STAT2 deficiency is relatively narrow, although characterized by incomplete penetrance. In addition, we report the first successful use of a Jakinib in a patient with a JAK–STAT defect and HLH, highlighting the complexity of the regulation of the type I IFN pathway and the need for more studies in order to offer these patients better therapeutic options.

## Materials and methods

### Inclusion of patients

Patients were included through referral by the treating physicians upon clinical presentation and upon identification of a *STAT2* variant in the whole-exome sequencing analysis. All patients or their legal guardians gave consent for participation in the study. The study was approved by the Ethical Research Committee of Leuven University Hospitals (study number S60905). Informed consent was obtained in France in accordance with local regulations and a human-subjects research protocol approved by the institutional review board of the Institut National de la Santé et de la Recherche Médicale (INSERM). Approval was obtained from the French Ethics Committee (Comité de Protection des Personnes), the French National Agency for Medicine and Health Product Safety, and INSERM in Paris (protocol C10-13). The data were collected via a case record form.

### Whole-exome sequencing

For P1 and P2: Whole-exome sequencing was performed as previously described (36). In brief, exome capture was performed with SureSelect Human All Exon 50 Mb kit (Agilent Technologies). Paired-end sequencing was performed on an Illumina HiSeq 2000 (Illumina), generating 100-base reads. The reads were then mapped onto the human reference genome (GRCh38/hg38) with the Burrows-Wheeler Aligner (37). Downstream processing was performed with the Genome Analysis Toolkit (GATK) according to documented best practice. All variants were then filtered and annotated with in-house developed software.

For P3: Genomic DNA was isolated by phenol-chloroform extraction from peripheral blood cells or primary fibroblasts from the patient. DNA (3 µg) was sheared with a Covaris S2 Ultrasonicator (Covaris). An adapter-ligated library was prepared with the TruSeq DNA Sample Prep Kit (Illumina). Exome capture was done using the SureSelect Human All Exon 50 Mb kit (Agilent Technologies). Paired-end sequencing was done on an Illumina HiSeq 2000 (Illumina), generating 100-base reads. The sequences were aligned with the human genome reference sequence (hg19 build), with the Burrows-Wheeler aligner (v.0.7.12). Downstream processing was done using GATK (v.3.4), SAMtools (v.1.0) and Picard Tools (http://picard.sourceforge.net; v.1.92). Substitution and insertion or deletion (indel) calls were made using a GATK unified genotyper and GATK IndelGenotyperV2, respectively. All calls with a Phred-scaled single-nucleotide polymorphism quality of up to and including 20 and a read coverage of 2 or less were filtered out. All variants were annotated with annotation software developed in-house.

### Sanger sequencing

The *STAT2* gene was sequenced in PBMCs of P3 as well as in fibroblasts of P1, P2, and their mother. Sanger sequencing was performed on an ABI 3730 XL Genetic Analyzer (Applied Biosystems) at the LGC Genomics Facility in Berlin, Germany. The regions of interest in Exon17 (P1 and P2) as well as Intron9 (P3) were amplified using the following primers: 5′-UM13-CTGCAGCTAGAAACATCA-3′ and 5′-UM13-AAAAGGAAATCTGTACCGAA-3′ (P1 and P2); 5′-UM13-GGAGAAAGGAGGTGCTGGATG-3′ and 5′-UM13-AACTTCCGGAAGCCTTGTAAT-3′ (P3).

Sequencing was performed with the universal UM13 primers. A control sample of an unrelated control was included for both sequencing runs.

Variants ClinVar accession numbers: SCV007595874 and SCV007595875.

### PBMCs

Frozen PBMCs of P3 were available for analysis. After defrosting, 1 × 106 cells were used for the Sanger Sequencing (above), and a whole-cell extract was made in 100 µl NP40 buffer supplemented with protease inhibitors.

### T cell blasts

For P3 and a healthy control, T cell blasts were generated as follows: PBMCs were isolated by Ficoll-Hypaque density centrifugation (Amersham-Pharmacia-Biotech). For T-blast induction, PBMCs were cultured in ImmunoCult-XF T Cell Expansion Medium (STEMCELL) in the presence of ImmunoCultTM Human CD3/CD28/CD2 T cell activator (12.5 μl/ml) and human recombinant IL-2 (100 ng/ml, Novartis).

### Cell culture

Primary fibroblasts were cultured in DMEM/F-12 (1:1) containing L-glutamine and HEPES supplemented with 10% FCS and 1% Pen-Strep and 0.1% normacin. Cells were incubated at 37°C under the atmosphere containing 5% CO_2_. When the cells reached 80% confluency, the cells were trypsinized and split 1:4.

Fibrosarcoma cells deficient for STAT2 (U6A, kindly gifted by Prof. S. Boisson-Dupuis, Human Genetics of Infectious Diseases Laboratory, Rockefeller University, New York, NY, USA) were cultured in medium DMEM (1×) + GlutaMax-1 (cat #61965-026; Thermo Fisher Scientific) complemented with 10% FCS (cat #51818-500), Penicillin (100 IU/ml), and Streptomycin (100 µg/ml) (cat #15140-122; Thermo Fisher Scientific).

### pJet cloning

The splice variant identified in P3 was analyzed via the CloneJET PCR Cloning Kit (cat #K1232; Thermo Fisher Scientific) following the manufacturer’s instructions. In brief, the isolated RNA of P3 was reverse transcribed with Superscript Vilo cDNA synthesis kit (Thermo Fisher Scientific). The STAT2 gene of the relevant fragment was amplified as described above. Afterward, the amplified fragments were cloned into the pJET 1.2/blunt cloning vector and amplified in heat shock–transformed *Escherichia coli* (#C3040H; New England Biolabs). Purification of 182 clones was performed with the QIAprep Spin Miniprep kit (#27104; Qiagen) according to the manufacturer’s instructions. The sequences inserted were determined by Sanger sequencing with the manufacturers primers: 5′-CGA​CTC​ACT​ATA​GGG​AGA​GCG​GC-3′.

### Plasmid cloning

The coding sequence for *STAT2* (NM_005419.4) was cloned into an untagged pCMV6 vector (OriGene). The Q5 Site-directed Mutagenesis Kit (New England Biolabs) was used to generate the indicated *STAT2* variants. All constructs were re-sequenced (LGC Genomics) to ensure that no adventitious mutations were generated during cloning. The used primer sets are displayed in Table S1. In addition, each construct was expressed in HEK293T cells and analyzed via immunoblotting to ensure no damage to the plasmid’s backbone.

### Overexpression of STAT2 plasmids and immunoblotting

HEK293T- or STAT2-deficient U6A Fibrosarcoma cells were transfected with 100 ng of WT or mutant plasmid. Proteins of primary fibroblasts, PBMCs, and transfected cells were extracted in NP40 buffer supplemented with protein inhibitors. The lysates were run on 4–12% Tris-glycerine SDS-PAGE (Invitrogen), and the resulting bands were transferred to a polyvinyl difluoride membrane with 0.45-µm pores (Thermo Fisher Scientific), which was blocked with 5% bovine serum albumin in Tris-buffered saline. It was probed with unconjugated primary antibodies: STAT1 (sc-464; Santa Cruz Biotechnology, 1/500), pSTAT1 (9167S [Tyr701], Cell Signaling Technology 1/400), STAT2 (sc-514193; Santa Cruz Biotechnology, 1/400), pSTAT2 (88410; Cell Signaling Technology, 1/500), and HRP-conjugated secondary antibodies (goat anti-mouse [71045-3; Merck Life Science, 1/10,000], and mouse anti-rabbit [sc-2357; Sant Cruz Biotechnology 1/10,000]). An anti–β-actin antibody (Sigma-Aldrich, 1/10,000) was used as a loading control. SuperSignal West Pico Plus chemiluminescent substrate and Pierce ECL western blotting substrate (Thermo Fisher Scientific) were used to visualize HRP activity. Chemiluminescent signals were detected with a Bio-Rad Imager, and Image Lab 6.0.1 software was used for analysis.

### RNA analysis

RNA was extracted from primary fibroblasts (P1 and P2), frozen PBMCs (P3), or STAT2-deficient U6A fibrosarcoma cells (overexpression) that were collected in Trizol Reagent (Ambion). cDNA was generated with the SuperScript VILO cDNA synthesis kit (Thermo Fisher Scientific) according to the manufacturer’s protocols. qPCR for STAT2 was performed using SsoAdvanced SYBR Green Supermix (Bio-Rad) with a QuantStudio Real-Time PCR System (Thermo Fisher Scientific). The results are expressed according to the ΔCt method, with GAPDH as the housekeeping gene.

### RT-qPCR

The induction of IFN genes was measured in patient derived primary fibroblasts of P1 and P2 as well as in the overexpression system of STAT2 deficient fibrosarcoma cells previously transfected with STAT2 wild-type and mutant plasmids as indicated. After transfection cells were rested for 48 h and stimulated with 10,000 U/ml IFN-α2A (cat #H6041; Merck), 1,000 U/ml IFN-γ (R&D Systems) for the indicated time points. Trizol was added to the cells and RNA was extracted as well as cDNA generated (see RNA analysis). The upregulation of IFN-induced genes, *RSAD2*, *ISG15*, *Mx1*, *IFIT1*, *GBP1*, and *USP18* were analyzed via RT-qPCR. The used primer sets are displayed in Table S2. The results are displayed according to the ΔΔCt method, with GAPDH as the housekeeping gene. Data are normalized to the healthy control/WT conditions.

### FACS analysis

For measurement of the total cell expression of STAT2, control or patient PBMCs or T-blast cells were plated in 96-well plates, at a density of 5 × 105 cells per well, and surface-stained with D3 PE, CD14 FITC, and then permeabilized and stained intracellularly with pSTAT2 AF647 or pSTAT1 AF647 antibodies. The cells were then washed twice with PBS and analyzed by flow cytometry. Data were acquired on a Gallios flow cytometer, and the results were analyzed with FlowJo (Tree Star).

### Online supplemental material

Fig. S1 shows clinical presentation of the patients; and Fig. S2 shows expression of phosphorylated STAT2 (pSTAT2), STAT2, pSTAT1, and STAT1 in lymphocytes derived from P3 and two healthy controls. Table S1 shows primers for site-directed mutagenesis; Table S2 shows primers for qPCR; and Table S3 shows list of variants identified after analysis of the WES of the P1 and P2.

## Supplementary Material

Table S1 shows primers for site-directed mutagenesis.

Table S2 shows primers for qPCR.

Table S3 shows list of variants identified after analysis of the WES of the P1 and P2.

SourceData F2 is the source file for Fig. 2.

SourceData F3 is the source file for Fig. 3.

## Data Availability

The data underlying the figures are available in the published article and its online supplemental material.
