# Supplementary material for: Two novel kindreds with autosomal recessive STAT2 deficiency
Source: J Hum Immun. 2026 Jun 30;2(5):e20260037. doi: 10.70962/jhi.20260037 (PMC13317486; doi:10.70962/jhi.20260037)
Supplement: Table S1 — shows primers for site-directed mutagenesis. [file jhi_20260037_tables1.docx]

Supplemental Table 1: Primers for site-directed mutagenesis.

| Patient | Variant | Sequence |
| --- | --- | --- |
| P1 + P2 | K490Qfs*41 | F: CAAGGCCCCCTGGAGCTTG |
| frameshift mutation |  | R: GGGGGGGTTGGAGAAGAAC |
| P3 | Deletion exon 9 | F: AGCCTTTGTGGTAGAAAC |
| Splice variant |  | R: CATGTCTCCAGCTGTTCC |
| P3 | Insertion intron 9 | F: GGGAAAGAAGGAACAAGGGAAGCCAAGCCTTTGTGGTAGAAACC |
| Splice variant |  | R: CCAGGGTTCCTGCCTGGCCTCTAGACCTGTGGAGCAGACGCTGT |
|  |  | Second step: |
|  |  | F: TCTCTCCACACCTCTCTCTCCTCAGAGCCTTTGTGGTAGAAAC |
|  |  | R: ATATATAGCTCAGTATCTGTAAGAATGGCTTCCCTTGTTCCTT |
| P3 | Insertion intron 8+47 | F: GTCCTCGCCATGCGCTGTGATTTGTTCACAGCTGGAGCAAAG |
| Splice variant |  | R: GAGGGTTGGGGTGGTACCTCTCACCATGTCTCCAGCTGTTCC |
