# Supplementary material for: Two novel kindreds with autosomal recessive STAT2 deficiency
Source: J Hum Immun. 2026 Jun 30;2(5):e20260037. doi: 10.70962/jhi.20260037 (PMC13317486; doi:10.70962/jhi.20260037)
Supplement: Table S2 — shows primers for qPCR. [file jhi_20260037_tables2.docx]

Supplemental Table 2: Primers for qPCR.

| Gene | Forward Primer | Reverse Primer |
| --- | --- | --- |
| GAPDH | 5’-GTC TCC TCT GAC TTC AAC AGC G-3’ | 5’-ACC ACC CTG TTG CTG TAG CCA A-3’ |
| STAT2 | 5’-AGA AAG TTA ATC TCC AGG AAC GG-3’ | 5’- CCT AGT TCC AGC TCT AAT GAC TCC-3’ |
| ISG15 | 5’-GGT GGA CAA ATG CGA CGA ACC TC-3’ | 5’-CAC ACC CTC CAG CCC GCT CA-3’ |
| IFIT1 | 5’-GGT GGA CAA ATG CGA CGA ACC TC-3’ | 5’-AAT TCA ATC TGA TCC AAG AC-3’ |
| RSAD2 | 5’-GCG TCA ACT ATC ACT TCA CTC-3’ | 5’-CAG GTA TTC TCC CCG GTC T-3’ |
| Mx1 | 5’-GGC TGT TTA CCA GAC TCC GAC A-3’ | 5’-CAC AAA GCC TGG CAG CTC TCT A-3’ |
| GBP1 | 5’-CTC CAC GGT GCA GTC TCA- 3’ | 5’-CAT TCT GGT TGT CAC CCT-3’ |
| USP18 | 5’-TCA TGG CCT GGT TGG TTT ACA CA-3’ | 5’-TGG GCA CCG TGA TCC TCT-3’ |
