# Supplementary material for: Two novel kindreds with autosomal recessive STAT2 deficiency
Source: J Hum Immun. 2026 Jun 30;2(5):e20260037. doi: 10.70962/jhi.20260037 (PMC13317486; doi:10.70962/jhi.20260037)
Supplement: Table S3 — shows list of variants identified after analysis of the WES of the P1 and P2. [file jhi_20260037_tables3.docx]

Supplemental Table 3. List of variants identified after analysis of the WES of the P1 and P2

| Chromosome | Genomic DNA Position (GRCh38) | DNA ID | Reference Allele | Alternative Allele | Predicted Amino  Acid Change | Gene |
| --- | --- | --- | --- | --- | --- | --- |
| 11 | 117222691 | rs143659874 | C | A | Pro127His | CEP164 |
| 12 | 56349032 | - | T | TG | Lys490GlnfsTer41 | STAT2 |
| 12 | 69693669 | rs184815371 | A | G | - | BEST3 |
| 15 | 82345109 | rs200247767 | A | G | Cys251Arg | GOLGA6L10 |
| 16 | 11770900 | rs150823676 | T | C | Ile331Val | ZC3H7A |
| 3 | 195779038 | rs377017763 | C | T | Ser4181Asn | MUC4 |
