# Supplementary material for: Two novel kindreds with autosomal recessive STAT2 deficiency
Source: J Hum Immun. 2026 Jun 30;2(5):e20260037. doi: 10.70962/jhi.20260037 (PMC13317486; doi:10.70962/jhi.20260037)

Figure2A: Immunoblot of STAT2 in PBMCs of P3

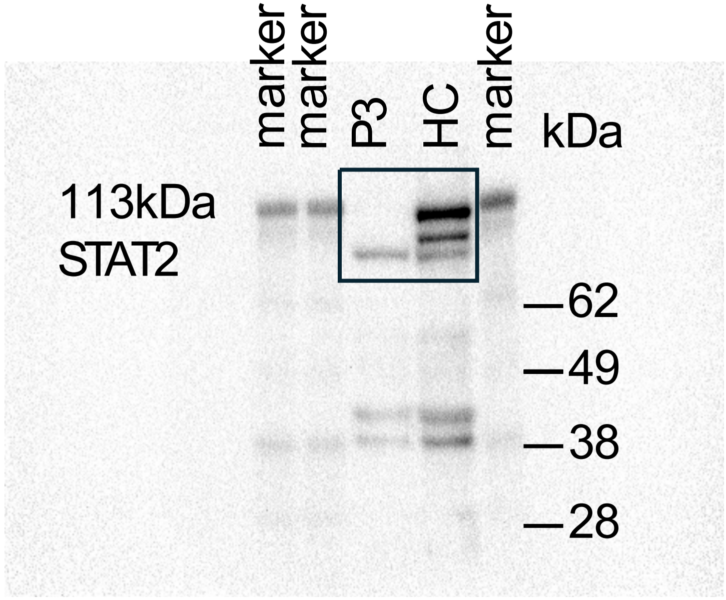

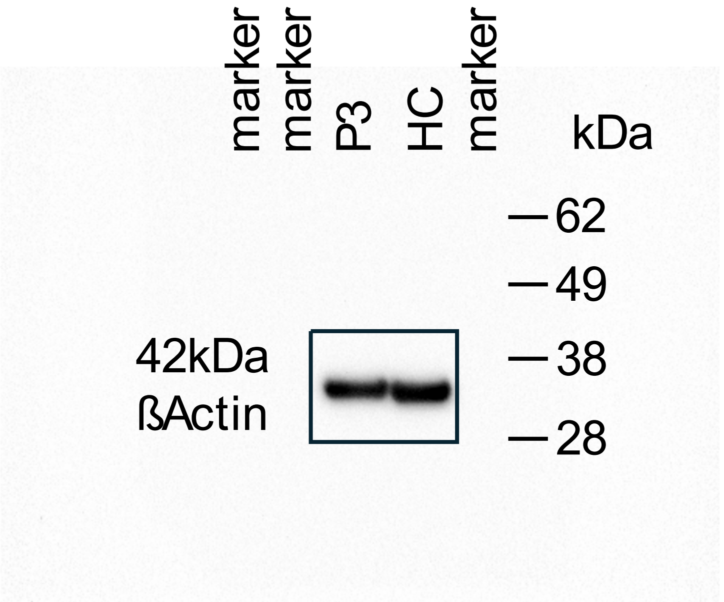

Figure2D: Immunoblot of STAT2 in HEK293T cells expressing different STAT2 variants

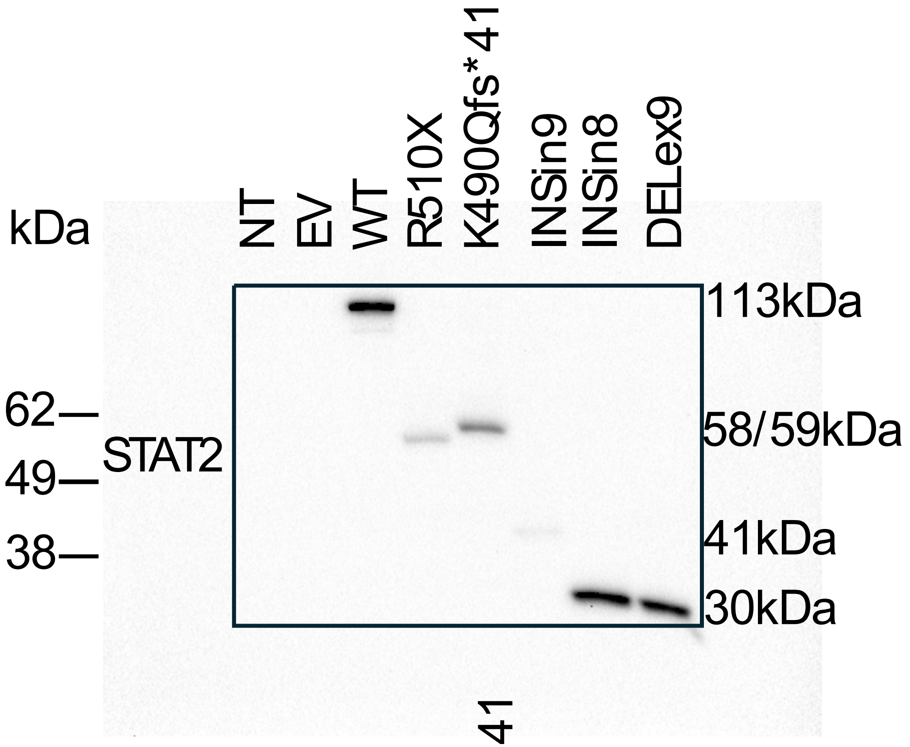

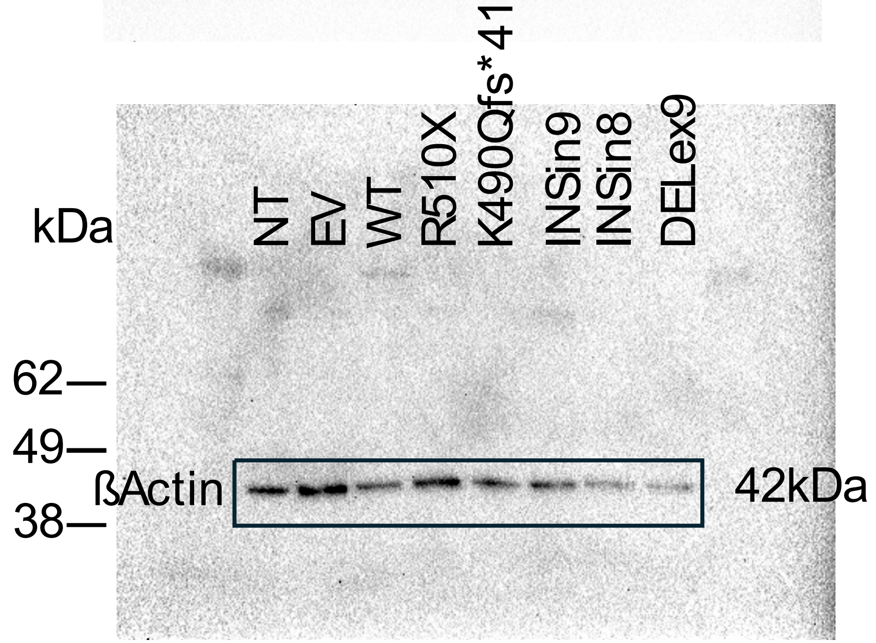

Supplement: SourceData F2 — is the source file for Fig. 2. [file jhi_20260037_sourcedataf2.pdf]
