# Supplementary material for: Two novel kindreds with autosomal recessive STAT2 deficiency
Source: J Hum Immun. 2026 Jun 30;2(5):e20260037. doi: 10.70962/jhi.20260037 (PMC13317486; doi:10.70962/jhi.20260037)

SourceData3A: Immunoblots of whole cell extracts from primary fibroblasts of P1 and P2, their mother (I.2), and three healthy controls (HC).

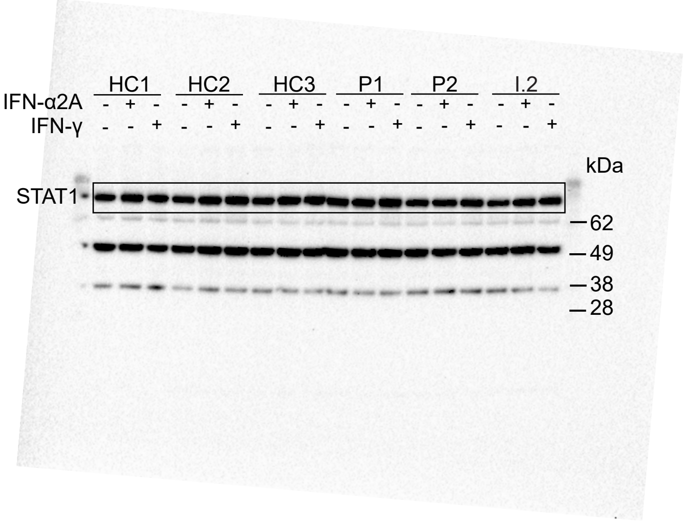

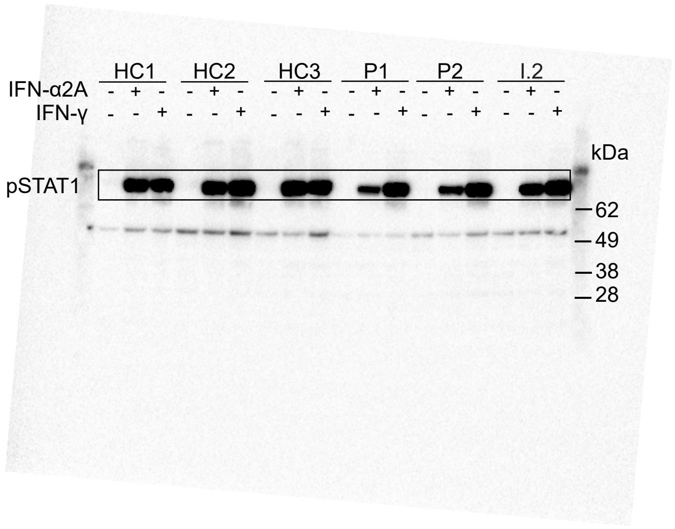

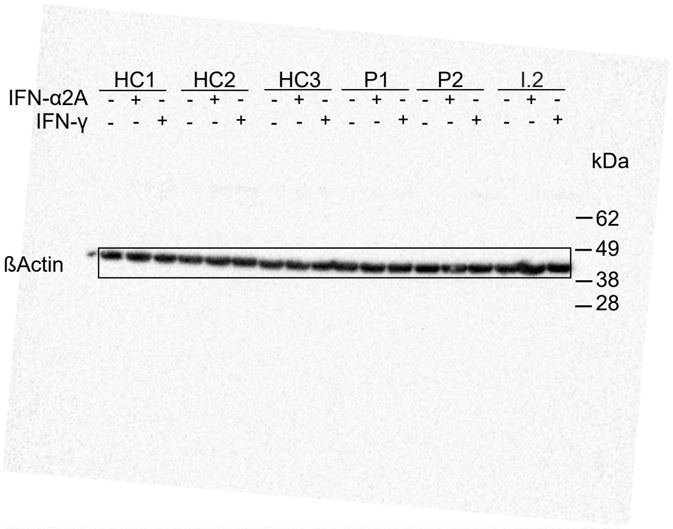

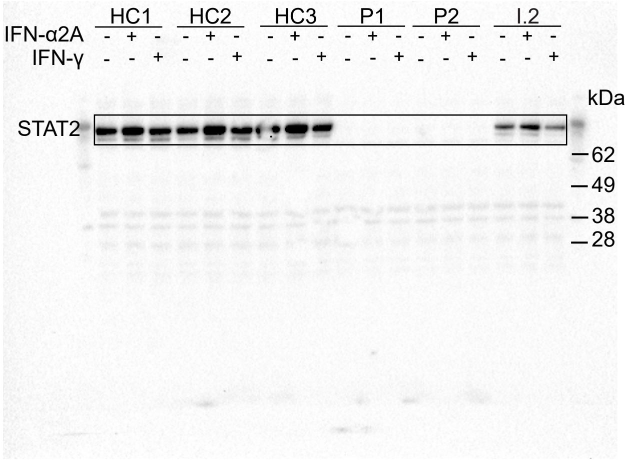

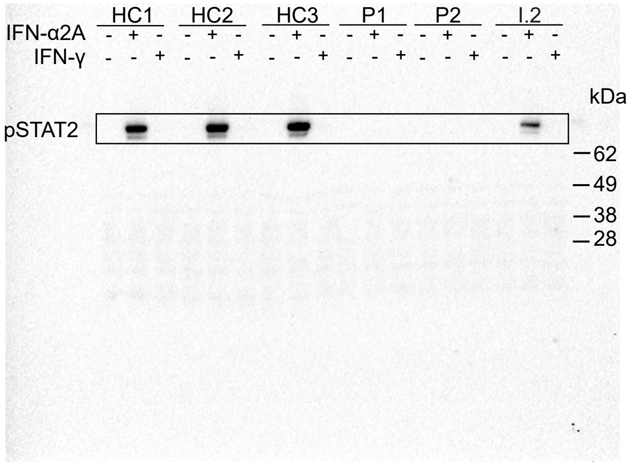

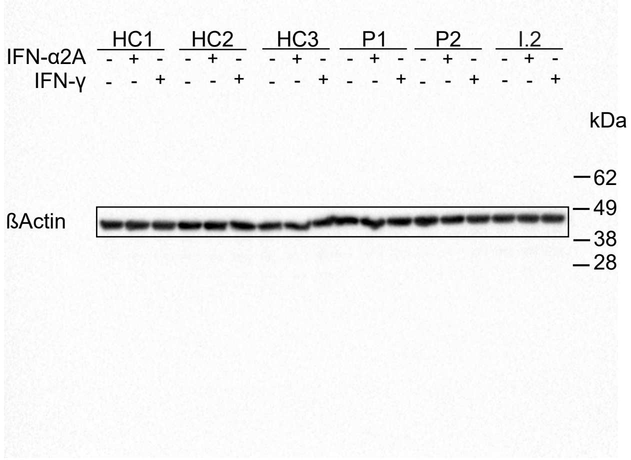

SourceData3B: Immunoblots of whole cell extracts from HEK293T cells transfected with STAT2 variants.

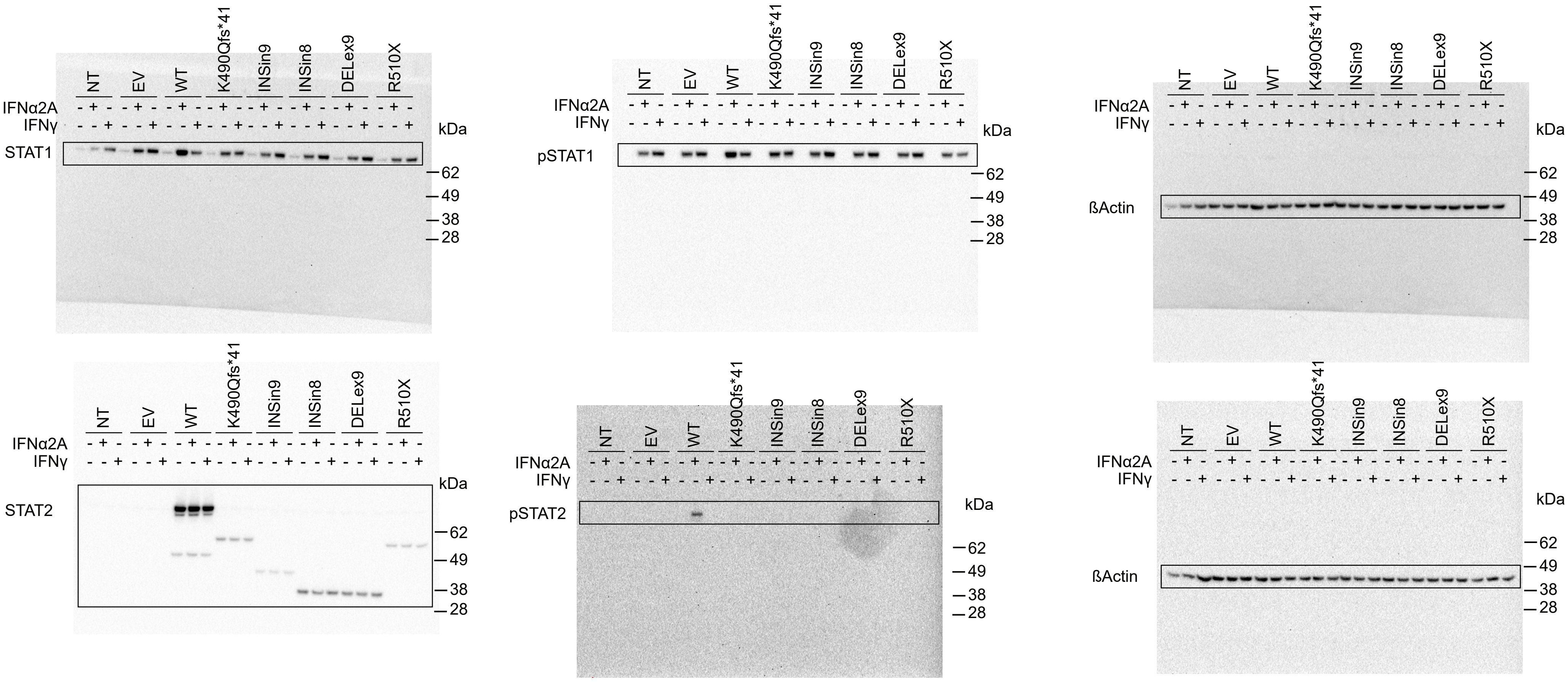

Supplement: SourceData F3 — is the source file for Fig. 3. [file jhi_20260037_sourcedataf3.pdf]
